# Unemployed + Sick = More Deserving? A Survey Experiment on How the Medicalization of Unemployment Affects Public Opinion

**DOI:** 10.3389/fsoc.2022.738397

**Published:** 2022-05-06

**Authors:** Philipp Linden, Nadine Reibling

**Affiliations:** Department of Social Sciences, University of Siegen, Siegen, Germany

**Keywords:** medicalization, social policy, social legitimacy, deservingness, unemployment, sickness, factorial survey, vignette study

## Abstract

The literature on the social legitimacy of welfare benefits has shown that sick persons are perceived more deserving than unemployed individuals. However, these studies examine sick and unemployed persons as distinct groups, while unemployment and sickness are in fact strongly related. Policymakers across Europe have been increasingly concerned with discouraging a medicalization of unemployment and activating sick unemployed persons. Therefore, it is crucial to understand welfare attitudes toward this group. Using a factorial survey fielded with a representative sample of German-speaking adults (N=2,621), we investigate how sickness affects attitudes toward a hypothetical unemployed person on three dimensions: benefit levels, conditions, and sanctions. Respondents allocated similar benefit levels to unemployed persons regardless of whether they have an illness. Yet, they were more hesitant to apply existing conditions (e.g., active job search, job training) or sanction benefits when the unemployed person was also sick. This is except for conditions that tie benefits to obligatory health services (back training or psychological counseling) which was supported by the majority of respondents. Our research shows that the German public is not more generous and only partially more lenient toward sick unemployed persons as there is strong support for conditions targeted at overcoming ill health for this group. The findings underscore that sickness matters for how unemployed persons are perceived, but the impact varies across different dimensions of welfare attitudes.

## Introduction

Sick individuals are generally considered more deserving by the public than persons who are unemployed (van Oorschot, 2000). However, many individuals belong to both groups because unemployment causes ill health and vice versa (Paul and Moser, 2009). For instance, one in four unemployed persons in Germany indicate to have a longstanding sickness like a chronic condition or disability, and almost half express poor health in general (Bambra and Eikemo, 2009). As access to disability benefits has been tightened across Europe, many advanced welfare states are challenged how to address the large group of unemployed persons with health limitations within the existing activation regimes (Holmqvist, 2010; Alanko and Outinen, 2016). International organizations have argued that this group requires targeted services that meet their needs and welfare states have strengthened health-promoting and rehabilitative services for unemployed persons in recent years (OECD, 2010; European Network of Public Employment Services, 2020). In this context, it is crucial to investigate the public's perception of this group and the perceived legitimacy of the (planned) policy measures that address them. Therefore, this study investigates how attitudes toward welfare policies and their legitimacy (Brooks and Manza, 2007; Frederiksen, 2018) are affected if a person's unemployment is attributed to an illness.

We present two theoretical perspectives to examine the link between ill health and attitudes. First, we build on the literature on the social legitimacy of welfare benefits (van Oorschot et al., 2017) which has shown that the deservingness of specific target groups such as the unemployed are conditional on the characteristics of this group. Our study introduces health status as a characteristic of unemployed persons that we expect to shape public opinion. Many recent contributions in this literature have argued that welfare attitudes contain various dimensions and thus called for differentiated measurements that distinguish between the generosity of benefits and the conditions under which they are granted or revoked (Gallego and Marx, 2017; Buß, 2018; Naumann et al., 2020). In this study, we follow this approach and use benefit levels, conditions, and sanctions as three outcomes, because we expect that sickness has different implications for each dimension. Moreover, we argue that it is particularly important to explore how existing activation measures, but also potential policies for sick unemployed individuals are perceived by the general public.

To better understand the link between ill health and obligations, we propose medicalization theory as a second strand of literature that has investigated how a medical explanation and treatment of social problems affects attitudes and role expectation toward sick persons for decades (Zola, 1972; Conrad, 1992; Pattyn et al., 2013). Medicalization denotes to a process in which a problem is understood as a medical problem and/or treated by the medical system (Conrad, 2008). Recent work in this field has demonstrated that unemployment has been increasingly medicalized (Miles, 1987; Ford et al., 2000; Holmqvist, 2009). While this may partially reflect the better understanding of the disease burden among the unemployed, it has also been discussed as a reaction toward the activation turn (Knotz, 2012; van Kersbergen and Hemerijck, 2012): With tighter conditionality of benefits (Dwyer, 2008) a sickness or disability has become an increasingly important justification for accessing benefits or being relieved from obligations (Hansen et al., 2014; Wong, 2016; Roosma and Jeene, 2017). The medicalization literature has emphasized that a sickness relieves individuals from social role obligation which helps to justify inactivity and benefit receipt. Particularly with reference to work, however, the sick role is linked to social control around monitoring and overcoming the sickness state. Thus, we might expect that the public favors policy initiatives targeting the health of unemployed persons to improve their employability.

This study makes three unique contributions to the current literature. First, it investigates how attitudes toward recipient groups change when individuals are simultaneously unemployed and sick. Second, we utilize medicalization theory to provide a new perspective on welfare state attitudes that highlights that sickness is a socially constructed category. Finally, based on the medicalization perspective, we extend the current operationalizations of obligations by another form of social control: obligation to seek treatment (Zola, 1972; Freidson, 1988; Parsons, 1991; Conrad, 1992, 2008; Conrad and Schneider, 1992).

In line with the existing standard to understand the impact of characteristics on attitudes (Pattyn et al., 2013; van Oorschot et al., 2017; Schofield and Butterworth, 2018) we conducted an experimental vignette study with a representative sample in Germany. The development of the vignette was based on the existing regulations at the time of data collection (Social Code Book II and III). Income replacement for unemployment is organized through a contribution-based unemployment insurance system which pays around 60% of previous earnings up to 12 months and a means-tested minimum income insurance program. Access to unemployment insurance benefits is tied to previous employment and contribution and considered relatively strict by OECD standards (Venn, 2012). If a person is not entitled or has exhausted maximum duration of unemployment benefits, minimum-income benefits can be claimed. The standard benefit is €432 for single person. Access is means-tested. In both systems, benefit receipt is tied to certain conditions, e.g., actively looking for a job, participating in training measures, taking up any job. These conditions are considered very strict in international comparison (Venn, 2012). In the event, of non-compliance with regulations, gradual sanctions can be imposed up to a full cancellation of benefits. However, sanctions are considered rather mild in international comparison (Venn, 2012). Moreover, the system is currently under reform due to a Constitutional Court Ruling in November 2019 (after our data collection).

The aim of our study is 3-fold. First, we examined if a physical or mental health condition as a reason for unemployment affects respondents' perception of *deservingness* operationalized through the allocation of unemployment and minimum income benefits to a hypothetical male unemployed person (Aim 1). Second, we investigated how attitudes toward *social control* (attitudes toward obligations and sanctions) are affected by the presence of a mental or physical sickness (Aim 2). Finally, we examined if respondents would support an obligatory take-up of specific health services which is a possible policy measure or practice targeted at this group (Aim 3).

## Theoretical Background

The aim of this article is to explore how sickness affects welfare attitudes toward unemployment. Subsequently, we present the social legitimacy approach and medicalization theory as two complementary perspectives for deriving expectations on attitudes toward unemployed *and* sick individuals. We argue that how sickness affects attitudes toward unemployment depends upon the specific attitudinal dimension examined. We consider (a) attitudes toward *benefits* as measures of the social rights that the public considers adequate and (b) attitudes toward *conditions* and *sanctions* as perceptions of the social control measures deemed acceptable by the public.

### Attitudes Toward Social Rights: Benefit Levels as Measures of Deservingness

The literature on the social legitimacy of welfare aims to explain to what extent groups that are targeted by welfare state programs are considered deserving. Deservingness perceptions are theorized as based upon five criteria of the CARIN model (van Oorschot, 2000, 2006): (1) how much *control* the individual has over the reasons for the need of assistance, (2) what *need* actually exists and whether it is justifiable to claim it, (3) the level of *reciprocity*, whether an individual has contributed to society in the past or can be expected to contribute in the future, (4) the *identity* of and cultural distance to a welfare claimant (5) and the *attitudes* that can be attributed to the individual which help to overcome the need for assistance as quickly as possible. A well-corroborated finding in this literature is that sick individuals are markedly more deserving than unemployed persons (van Oorschot et al., 2017) and also rated more positively in terms of warmth (Schofield et al., 2021).

However, we argue that the existing evidence is limited in two respects. First, sick and unemployed individuals are treated as distinct groups, while in reality unemployment and sickness are highly correlated (Paul and Moser, 2009). Thus, it remains unclear whether the higher deservingness attributed to sick persons can be generalized to the situation when they are *also* unemployed. Secondly, existing studies which compare different types of target groups (such as sick and unemployed persons) have focused on general support measurements only, e.g., should the government be responsible for healthcare for the sick or provide a decent standard of living for the unemployed (Jensen and Petersen, 2017). This choice is useful for comparing different target groups because the type of benefits individuals receive is different for the sick (healthcare services) than for the unemployed (monetary benefits). Nevertheless, it means that we cannot infer from the existing studies how respondents judge these groups on other attitudinal dimensions, especially the conditions perceived acceptable for benefit receipt.

Still, the CARIN model of deservingness allows to develop our expectations on how sickness might affect attitudes in the context of unemployment. As a starting point, it is necessary to consider how sickness can be conceptualized within the model. It is well-established that neither all criteria are always relevant nor is one criterion generally more important than others, but rather the weights of criteria differ between individuals and contexts (de Vries, 2017). Within the context of sickness and disability (the lack of), *control* over one's health has generally been suggested as the most relevant criterion that explains why sick individuals are considered more deserving than other groups (de Swaan, 1988; van Oorschot, 2006). While sickness is often considered as a fateful state, unemployment is perceived to be at least partially under the control of individuals, thus leading to a lower perceived deservingness of this group. Therefore, individuals who are unemployed *and* sick may be considered as having low control. Previous research has shown that low control over one's unemployment status is associated with higher benefit levels (Buß, 2018) and thus we expect that sick unemployed persons are allocated benefit levels comparable to other groups with low control over their unemployment:

H1: Individuals, who become unemployed due to a sickness receive *similar* levels of benefits than individuals who are healthy but have also low control over losing their job.

### Social Control Perceptions: Attitudes Toward Obligations and Sanctions

Most work on deservingness perceptions has focused on the public's attitudes toward social rights. However, a second important dimension of deservingness are the social control measures institutionalized within the welfare state as obligations and sanctions (Meuleman et al., 2017). This dimension has gained importance with the turn toward activation leading to a stronger individualization of social rights (Knotz, 2012; van Gerven and Ossewaarde, 2012). By placing institutional constraints on labor market policy reforms (Clasen and Clegg, 2006), this emphasized welfare conditionality (Clasen and Clegg, 2007; Dwyer, 2008).

Studies on attitudes on obligations and sanctions show that the support for these measures is quite substantial (Houtman, 1997; Buß, 2018; Naumann et al., 2020). However, obligations and sanctions are perceived differently depending on the characteristics of the described unemployed person. Again, control over re-employment chances is an important criterion that has been considered here as the reason why there is less support for conditions and sanctions for older persons, persons with family obligations and disabled persons (Roosma and Jeene, 2017; Buß, 2018; Naumann et al., 2020). In this vein, we expect that unemployed persons who are also sick are perceived as having less control over re-employment, because they may not be able to take certain jobs and they are discriminated by employers (Hipes et al., 2016). Moreover, certain conditions such as regional mobility may seem inadequate if individuals are sick (Dwyer, 2002) and require healthcare (and in the case of mental sickness a stable social environment). Finally, sick unemployed persons should be perceived as more in need as they require healthcare and are limited in their activities. Thus, we presume that sanctions should seem less appropriate for this group of unemployed persons:

H2: Unemployed individuals, who are sick, receive lower non-medical obligations and are sanctioned less compared to the other unemployed persons.

So far, we have shown how we can develop expectations on the role of sickness in the context of different dimensions of unemployment attitudes based the social legitimacy literature. In this literature, sickness has generally been perceived as a category with a substantial gain in deservingness (Jensen and Petersen, 2017; van Oorschot et al., 2017). We believe that this perspective can be complemented a different perspective on the link between unemployment and sickness: *medicalization theory*.

Conrad (2008) denotes medicalization as process by which social phenomena become interpreted and/or treated as medical problems (for example by medical professionals or medical interventions). Over the past two decades, a number of scholars have suggested that new insights can be gained on the link between unemployment and sickness by applying a medicalization perspective (Holmqvist, 2009; Buffel et al., 2015; Wong, 2016), because the role of medicine in understanding and dealing with unemployment has increased with the turn to activation (Schram, 2000; Holmqvist, 2009). Medicalization theory assumes that sickness and disability are socially constructed categories (Dwyer, 2002). Thus, they depend on existing institutional regulations are actively used by actors, e.g., to explain failing reintegration into the labor market (Holmqvist, 2010; O'Brien, 2015). While existing research has used medicalization theory to understand variation in sickness and disability of unemployed persons, medicalization theory has also important implications for attitudes toward sick unemployed persons. First, as Parsons (1991) already suggested the main function of the sick role is to legitimize that normal role obligations cannot be fulfilled. Thus, medicalization theory leads us to the expectations formulated in *H2*. Second, medicalization theory highlights, that obligations of sick persons are not generally lower; they rather face different obligations (Zola, 1972; Conrad, 2008):

“[…] the sick role involves a *relative* legitimacy, that is so long as there is an implied ‘agreement’ to ‘pay the price’ in accepting certain disabilities and the obligation to get well” (Parsons, 1991: p. 211).

Hence, we expect that lower public support for existing obligations in activation system coexist with supportive attitudes toward the obligation to seek treatment and actively work toward recovery:

H3: For the unemployed with an illness, support for obligations aimed at recovery will be stronger than for other non-medical obligations (i.e., active job search, work obligation).

## Methods

### Experimental Survey Design

Factorial surveys are widely used for assessing attitudes in both medicalization and welfare deservingness research (e.g., Pattyn et al., 2013; van Oorschot et al., 2017). The appeal of this approach lies in the combination of causal effect identification through the experimental design with the stronger external validity of survey research when using large, representative samples (Jasso, 2006; Atzmüller and Steiner, 2010; Aguinis and Bradley, 2014; Dülmer, 2016). Moreover, such a design is appropriate, when experimental manipulations are difficult, perhaps impossible (Rettinger and Kramer, 2009), ethically questionable (Graeff et al., 2014) or when respondents are to be questioned about sensitive topics (Aguinis and Bradley, 2014). In this context one might also argue that artificial variation in a case description might not reflect the outcomes of manipulation in a real-world experiment. However, vignette variations provide at least “a good substitute for similar manipulations in the real world” (Rettinger and Kramer, 2009, p. 297). They thus enable the representation of sensitive and complex phenomena in easily understandable and realistic situation descriptions (Auspurg and Hinz, 2015) and “produce a high level of response consistency during ordinary response times” (Sauer et al., 2011, p. 98)

The design of the vignettes and the following items were based on the study by Buß (2018). In our survey, we adapted and developed the wording of vignettes and instruments (see Supplementary Material S1 for an overview of vignette texts, questions, provided information and anchors). In the experiment we varied five dimensions with two to four levels for the description of the person and another dimension with two levels for the misbehavior leading to a sanction (Table 1).

**Table 1:** Vignette dimensions, levels, and coding.

**Vignette dimension**	**Levels**	**Coding**	**CARIN criterion**
Name of unemployed person	Mr. Bergmann|Mr. Yildirim	1|2	Identity
Age of unemployed person	25|40|60	1|2|3	Reciprocity
Reason for unemployment	Social—Personal misconduct	1	Control
	Economic—Bankruptcy of employer	2	
	Medical—Chronic backpain	3	
	Psychological—Depression	4	
Family status	Single|Married, no kids| Married and 3-year-old child	1|2|3	Need
Motivation	Less|Very	1|2	Attitude
Missed appointments	1st time|2nd time	1|2	Attitude

Each interviewee evaluated one vignette of a male person who was employed by a company in the last 2 years before losing his job. Respondents might have a different perspective on unemployed women due to gender norms, but since this was not the focus of the present analysis, we limited the description to men with a working record that entitles him to full unemployment benefits.

We operationalized the medicalization of unemployment, by providing a corresponding condition (chronic back pain/depression) as reason for the lay-off.^1^ To identify the specific effect of sickness on deservingness perception, we used two additional explanations which vary the extent of control over the unemployment: Bankruptcy of the employer (low) and personal misconduct (high). We controlled for alternative explanatory factors for differences in deservingness perceptions, outlined in the concept of CARIN-criteria (van Oorschot et al., 2017) by varying individuals' ethnicity (identity), age (reciprocity), family status (need) and the motivation to find a new job (attitude). An example vignette text reads as follows (bold text indicates the varied vignette dimensions, not bolded within the survey):

**Mr. Bergman** is **40** years old. He was employed by a company for the past two years but was dismissed due to **his chronic back pain** and has now been unemployed for one month. Since then, he has been trying **very** hard to find a new job. He is **married and has a 3-year-old child**.

After reading the vignette, we asked respondents a series of questions to cover the broad spectrum of rights and responsibilities within the German social security system. First, participants should allocate unemployment benefits. In line with current legal requirements, we provided the information that former employees on average, receive 60% of their last net income as benefits to ensure that respondents' allocation is not biased by differences in knowledge of regulations. Participants could choose between 0 and 100% of previous earnings. In a second step, we told them that the same person did not find a new job after 1 year and had to apply for minimum income benefits. Again, we implemented an anchor—based on the existing legislation—which states that a single person without children is legally entitled to about €400 per month, independent of previous earnings, plus their individual rent and heating costs. Allocation of benefits was possible between €0 and €1,000. Hereafter, we asked which obligations the described person must fulfill to receive the full amount of benefits. Respondents had the option to either allocate benefits unconditionally or to tie any of the actual FEA instruments (for example to engage in job trainings) to the receipt. If the job-loss happened due to a medical/psychological condition, we additionally offered respondents a compulsory rehabilitation measure: back training or psychological counseling. Finally, we told them that the FEA is authorized to cut benefits if the described individual misses an appointment with the responsible consultant. The corresponding vignette text states the following:

**Mr. Bergman** has failed to show up for an appointment at the employment agency for the **first** time without excuse. In this case, the employment agency has the option of reducing his or her remuneration for a period of three months.

We then asked the respondents to indicate by how much they would reduce the benefits (0–100%)^2^ in the event of non-compliance.

### Data and Participants

The factorial web-based survey (CAWI) was fielded within the online panel of YouGov Germany during September 2019. A quota sampling created a subset of 2,837 sample attendees invited to our study. Two hundred and sixteen persons (8%) canceled the interview early, leading to an analysis sample of *N* = 2,621 that is representative for the German adult population on key variables like gender, education, residence, and age (Supplementary Material S2). The median processing time of the survey was 14.12 min.^3^ Respondents were carefully debriefed^4^ at the end and received an incentive of 500 Tokens (€1) from YouGov.

The survey included questions about respondents' migration background, if they had worked in a job that is related to the concepts within the survey (medicine, psychology, or education), or if they had been unemployed in the last decade. Moreover, we requested information about their income in Euro as well as their political self-assessment and their actual health status.

Item-nonresponse ranged from almost none to 17.05% for income (Supplementary Material S3). We therefore estimated a multiple imputation by chained equations (MICE) regression model including survey weights with the *mi* commands in STATA 16 and used the aggregated values (N_Imputations_ = 20) of the full information sample for our analysis.

Nine in ten participants had no migration background and did not work in a survey-related job (Table 2). Two in three had no experience with unemployment in the last decade while six in ten indicated an income between €1,000 and €3,000. On average, participants assessed themselves politically slightly to the left (*M* = 4.75, *SE* = 1.99) and in a rather good state of health (*M* = 2.41, *SE* = 0.91).

**Table 2:** Descriptive statistics of respondent characteristics after MICE.

	**Descriptive statistics**	
**Categorical variables**	**Absolute**	**%**
Age		
18–39	731	27.89
40–60	1,028	39.22
60+	862	32.89
Region		
Western Germany	2,093	79.38
Eastern Germany	528	20.62
Migration background		
No	2,353	89.77
Yes	268	10.23
Experience in survey related profession		
No	2,391	91.22
Yes	230	8.78
Unemployed in last 10 years		
No	1,753	66.88
Yes	868	33.12
Income (in Euro)		
Below 500	272	10.38
500–999	411	15.68
1,000–1,999	1,063	40.56
2,000–2,999	629	24.00
3,000 and more	246	9.39
Self-reported health status		
Poor	80	3.05
Fair	299	11.41
Good	942	35.94
Very good	1,066	40.67
Excellent	234	8.93
Quasi-metric variables	**Mean**	**Standard error**
Age	48.53	0.363
Political self-assessment (0 = Left – 10 = Right)	4.75	0.185
Self-reported health status (0 = Poor – 4 = Excellent)	2.42	0.039

*Vignette study (N = 2,621), own weighted sample calculations*.

### Robustness Checks

First, we wanted to ensure that answers were not biased by socially desirable response patterns. Therefore, we used the KSE-G scale (Kemper et al., 2012) to measure social desirability by six items on two subscales (PQ+ as indicator of an overrating of positive characteristics & NQ- as indicator of underrating negative characteristics, see Supplementary Material S4). Respondents had the possibility to answer from 0 = “Doesn't apply at all” to 4 = “Applies completely” on statements like “It has happened that I have taken advantage of someone in the past” or “In an argument, I always remain objective and stick to the facts”. Compared to a representative population sample, our respondents overrate their positive characteristics slightly less as shown by lower means within the single items. The overall mean of the PQ+ subscale, however, shows no statistical difference between the two samples. Moreover, interviewees in our sample underrate their negative characteristics significantly less in both, the single items and within the overall mean of the NQ- subscale. Since these results imply a slight tendency toward the middle, we see no evidence for a bias toward social desirability, at least not more than in the overall population.

Second, since our vignette universe consists of N_Vignettes_ = 288, it was impossible for the respondents to answer all vignettes. We therefore randomly allocated each respondent to one specific vignette within the universe. Thus, our analysis based on the average of roughly 10 ratings per vignette, which cluster in one deck. We checked if the allocation to a specific deck had an influence on our findings. However, neither did the deck variable had a significant effect within any of the models, nor did it improve the overall fit [Unemployment benefits: LR χ^2^ (1) = 0.08, *p* = 0.772; Minimum income benefits: LR-χ^2^ (1) = 0.00, *p* = 0.991; Sanctions: LR χ^2^ (1) = 0.13, *p* = 0.723]. In addition, a mixed multi-level regression (ML) with deck as Level 2 variable does reveal a very low ICC in all three ML specifications (0.001–0.022) suggesting only weak correlations between respondents.

Finally, because of the factorial design, all factors were uncorrelated (Supplementary Material S5). Correlations between these factors and respondent characteristics were insignificant, except for four statistically significant but relatively weak correlations with a maximum of *r* < |0.058|.

### Analytical Strategy

We specified OLS regression models for unemployment benefits (in %), minimum income benefits (in Euro) and sanctions (in %) as dependent variables. To decompose the effects of different explanations of unemployment on the approval ratings to certain obligations, we additionally specified logistic regression models using the recommendation of FEA instruments as well as the medical/psychological intervention as dependent variables (0 = No vs. 1 = Yes). In all models we used an α = 0.05 for hypothesis testing and calibrated sample weights to compensate for deviations from the population distribution. While a factorial survey does not require controls due to the experimental design, a Likelihood-Ratio-Test (LR) indicates that they significantly improve the model fit [Unemployment benefits: LR χ^2^ (20) = 105.86, *p* < 0.001; Minimum income benefits: LR χ^2^ (20) = 128.75, *p* < 0.001; Sanctions: LR χ^2^ (20) = 218.72, *p* < 0.001]. Finally, we conducted an analysis with interaction terms between different vignette variables as well as between vignette and respondent variables but found no substantial effects.

## Results

### Deservingness Perceptions: Acceptance of Benefit Levels and Sanctions

**Figure 1:**
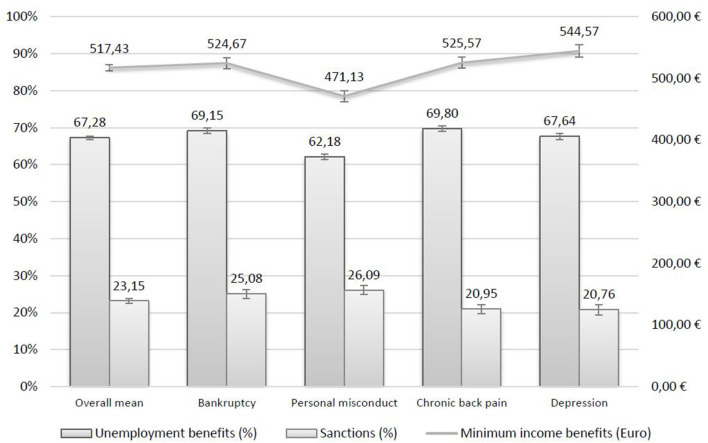
Allocation of unemployment benefits, minimum income benefits and sanctions with 95%-confidence intervals for different reasons of unemployment. Vignette study (*N* = 2,621), own weighted sample calculations. Bar heights (in %) and absolute values (in Euro) reflect predicted mean values of corresponding variables.

The reason for unemployment influenced to which extent respondents accepted benefits and sanctions (Figure 1). In comparison to a bankruptcy of the employer, respondents allocated 7% points (PP) less unemployment and €53 less minimum income benefits when individuals were laid off because of a personal misconduct. Unemployment caused by chronic back pain resulted in similar benefit levels compared with bankruptcy, whereas depression reduced unemployment benefit levels by 2 PP but increased minimum income benefit levels by almost €20. On average, respondents allocated 67% of previous earnings for unemployment benefits and €517 minimum income benefits, both exceeding the benefit levels of existing legislation for singles (60% and €400).

Three in four of our respondents accepted a cut of minimum income benefits by up to 23% on average if the described individuals did not follow the FEA rules. A medical or psychological reason for unemployment led to less acceptance of benefit cuts—around 21%—compared to 25% in the case of bankruptcy and 26% for personal misconduct.

Table 3 reports the results from the multivariate regressions of benefits and sanction regressed on the randomly assigned vignette dimensions and characteristics of the respondents. In comparison to a bankruptcy of the employer, respondents allocated significantly less benefits when individuals were laid off because of a personal misconduct. More striking, however, is the fact that unemployment caused by chronic back pain resulted in similar benefit levels compared with bankruptcy, whereas depression reduced unemployment benefit levels but increased minimum income benefit levels. However, respondents cut benefits significantly less, if the job-loss is explained by a health condition, while personal misconduct did not differ from bankruptcy in terms of sanctions.

**Table 3:** OLS regression of allocation of unemployment benefits, minimum income benefits and sanctions on vignette dimensions and respondent characteristics.

	**Unemployment benefits**	**Minimum income benefits**	**Sanctions**
**Vignette dimensions**			
Reason *(Ref.: Bankruptcy of employer)*			
Personal misconduct	−6.78^***^ [0.94]	−50.83^***^ [12.32]	1.03 [1.49]
Chronic back pain	0.79 [0.85]	3.45 [12.39]	−3.78^**^ [1.43]
Depression	−1.16 [0.96]	23.05 [12.94]	−4.32^**^ [1.40]
Name *(Ref.: Mr. Bergmann)*			
Mr. Yildirim	−6.42^***^ [0.65]	−64.15^***^ [8.78]	4.39^***^ [0.99]
Age *(Ref.: 25 Years)*			
40	0.88 [0.84]	20.37 [10.97]	−0.19 [1.26]
60	2.72^***^ [0.79]	40.35^***^ [10.79]	−2.77^*^ [1.19]
Family status *(Ref.: Single, no kids)*			
Married, no kids	0.53 [0.79]	34.46^***^ [10.28]	−0.37 [1.23]
Married, 3-year-old child	−0.40 [0.80]	60.76^***^ [11.07]	−0.82 [1.20]
Motivation *(Ref.: High)*			
Low	−4.65^***^ [0.66]	−51.59^***^ [8.85]	2.90^**^ [1.00]
Missed appointments *(Ref.: 1st* time*)*			
2nd time			11.66^***^ [1.00]
Respondent characteristics			
Gender *(Ref.: Male)*			
Female	1.03 [0.72]	−10.00 [9.40]	1.45 [1.05]
Education *(Ref.: Secondary level)*			
Primary level	−0.30 [1.67]	43.72^*^ [20.17]	0.23 [2.33]
Tertiary level	−1.53 [0.83]	2.58 [12.32]	−3.22^*^ [1.42]
Region *(Ref.: Western Germany)*			
Eastern Germany	0.17 [0.85]	−38.81^***^ [11.35]	0.74 [1.28]
Age *(Ref.: 40–60 Years)*			
18–39	−2.48^**^ [0.90]	−41.00^***^ [11.23]	2.75^*^ [1.33]
60+	−0.73 [0.73]	2.96 [10.65]	0.11 [1.20]
Migration background *(Ref.: No)*			
Yes	−1.05 [1.27]	8.96 [16.02]	2.93 [1.76]
Employed in survey-related job *(Ref.: No)*			
Yes	−0.32 [1.28]	11.92 [17.80]	−0.08 [2.01]
Unemployed in last 10 years *(Ref.: No)*			
Yes	2.53^**^ [0.83]	39.80^***^ [10.88]	−3.55^**^ [1.17]
Income in Euro *(Ref.: 1,000–1,999)*			
Below 500	−2.53^*^ [1.19]	−11.27 [16.81]	−1.25 [1.83]
500–999	−2.18 [1.14]	10.77 [13.67]	−1.97 [1.64]
2,000–2,999	0.17 [1.08]	4.15 [13.93]	−1.65 [1.52]
3,000 and more	−3.21^*^ [1.31]	16.01 [18.35]	0.26 [2.05]
Self-reported health (*Ref*.: Good)			
Poor	4.24^*^ [1.88]	−9.65 [29.47]	1.33 [3.36]
Fair	1.04 [1.33]	−0.54 [15.49]	−1.84 [1.73]
Very Good	1.12 [0.78]	−0.01 [10.57]	0.61 [1.16]
Excellent	2.08 [1.28]	−5.52 [19.01]	4.59^*^ [2.19]
Political self-assessment *(0 = Left – 10 = Right)*	−0.82^***^ [0.19]	−13.81^***^ [2.49]	2.43^***^ [0.27]
Constant	77.29^***^ [1.71]	598.00^***^ [22.86]	5.47^*^ [2.58]
Model diagnostics			
F-Test of overall significance	11.92	9.89	11.91
Df	27; 1730.2	27; 1806.7	28; 1515.9
Probability > F	<0.001	<0.001	<0.001
R^2^	0.115	0.102	0.080
Adjusted R^2^	0.106	0.092	0.070
Observations (Vignette evaluations)	2,621	2,621	2,621

*Vignette study (N = 2,621), own weighted sample calculations. Unstandardized weighted coefficients with standard errors in brackets. Ref. = Reference group*.

* *p < 0.05*,

** *p < 0.01*,

*** *p < 0.001 (two-tailed)*.

The other vignette dimensions also affected deservingness perceptions. Respondents allocated significantly less benefits and sanctioned more when the vignette person had a foreign name or showed a low motivation to find a new job. The 60-year-old individual was considered more deserving both in terms of benefit levels and sanctions. Being married and having a child both significantly increased minimum income levels, but not unemployment benefit levels or sanctions. In contrast, respondents sanctioned more if the described person had missed an appointment with the FEA advisor more than once.

### Social Control Perceptions: Support for Conditions to Receive Minimum Income Benefits

**Figure 2:**
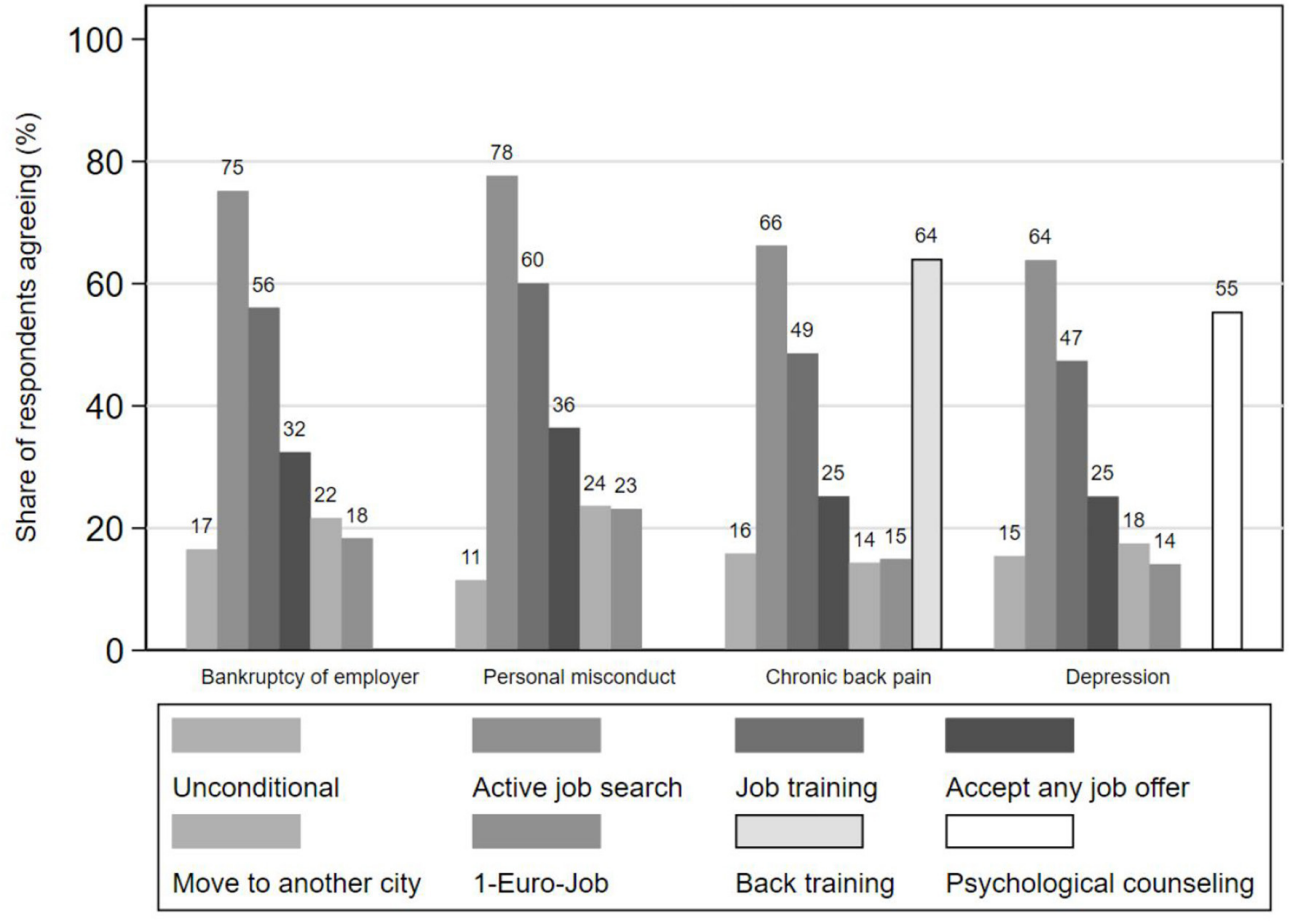
Expected behavior for receiving the full amount of minimum income benefits for different reasons of unemployment. Vignette study (*N* = 2,621), own weighted sample calculations.

A minority of one in six respondents agreed that the unemployed should receive minimum income benefits unconditionally (Figure 2). The majority, however, supported active job search and job training as conditions, about a third approved the instrument of accepting any job offer. Moving to another city or taking up a one-euro job were less supported conditions. If the vignette person has an illness, ratings differ: Support for all measures were between four and 11 percentage points lower compared to bankruptcy. However, most respondents supported sickness-specific conditions. 64% of respondents were willing to tie benefit receipt of an unemployed person with chronic back pain on obligatory back training; 55% supported obligatory counseling if the vignette person had a depression. Finally, respondents supported conditions slightly more strongly if the unemployed person was laid off due to personal misconduct compared with bankruptcy.

**Figure 3:**
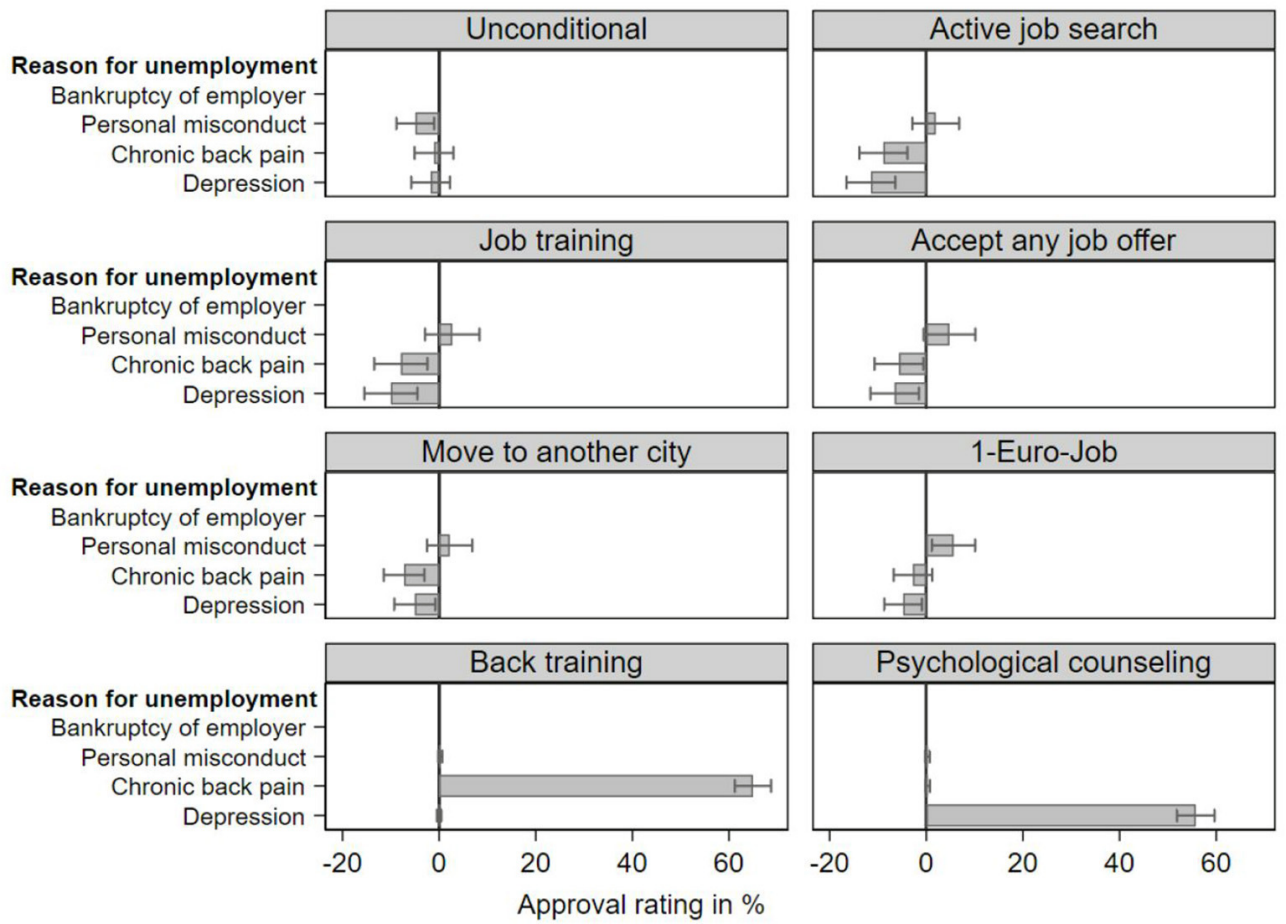
Multivariate logistic regression coefficients and 95% confidence intervals of approval ratings for obligations to receive full minimum income benefits for different reasons of unemployment. Vignette study (*N* = 2,621), own weighted sample calculations. Point estimates rescaled to percentage values. Model setup is the same as in the OLS-specification of benefits and sanctions (Table 3).

The multivariate results (Figure 3) supported the descriptive findings. Respondents were significantly less likely to support the various general conditions for minimum income benefits when a vignette person had an illness, except when taking up a one-euro-job for individuals with chronic back pain. However, support for sickness-specific interventions was large and significant. The small differences between personal misconduct and bankruptcy were mostly non-significant except for significantly stronger approval of taking a one-euro job for the unemployed person laid off due to personal misconduct. Moreover, respondents were significantly less likely to support unconditional benefit receipt for this group.

## Discussion

This study provides an examination of how attitudes toward unemployed persons change if they are also sick. Motivated by social legitimacy and medicalization theory, we explored how providing an illness as the reason for one's unemployment affects both *deservingness perceptions* and *attitudes toward social control measures*. By bringing together these two theories, we illustrated the dialectic implications of the sickness state for welfare attitudes: sickness is considered as a state of low control (“a matter of fate”) leading to empathy and more leniency toward this group. At the same time health is also perceived as the result of one's individual actions resulting in the expectation to actively participate in rehabilitative measures.

Starting from the literature of social legitimacy, we found that a person's control over a status of unemployment matters for respondents' assessment of deservingness but less toward social control perceptions. In terms of conditions and sanctions, attitudes are quite similar for the “healthy” unemployed persons with high and low control over their unemployment. Moreover, based on medicalization theory we broadened the scope of conditions considered to obligations addressing individual's health status. Our findings suggest that a sickness does affect how the public sees unemployed individuals and their need for assistance. To some extent this supports our expectations that the normal obligations of the unemployment regime are not applied in the same way to sick unemployed persons. Contrary to our expectations, however, sick unemployed are not considered as more deserving, but as less responsible. Thus, the medicalization of unemployment justifies, at least for part of the public, being more lenient and not fully applying the activation regime.

However, more than half of the respondents are willing to link the receipt of social benefits to mandatory health interventions. Thus, respondents' strong support for tying benefits to obligations does not diminish substantially even when unemployed individuals are also sick. We therefore conclude that a medicalization of unemployment does not ultimately lower expectations toward this group as has been often suggested by existing literature on social legitimacy (Petersen, 2012; Jensen and Petersen, 2017) but rather redirects them toward actively working toward better health.

### Policy Implications

Over the last three decades many European welfare states undertook major policy reforms with the aim to increase labor market participation of the inactive population. In 2003 Germany reformed its unemployment and minimum income system extending integration services to all non-employed persons, but also increasing the conditionality of benefit receipt under a mutual obligation framework. As early retirement options have been reduced and access to disability benefits is rather strict, a large number of sick unemployed persons is included in the activation system for the unemployed. A similar situation exists in other advanced welfare states (Martin, 2015). Despite the policy aim to activate this group, it has proven challenging (Eggs, 2013), since a large proportion of all long-term unemployed remain within the system and receive minimum income benefits for long periods of time (Duell et al., 2016). Our study shows that the German public generally agrees with the integration of sick unemployed persons in this system, as they do no allocate higher benefits to this group. While support for conditions and sanctions for this group have been significantly lower, our study shows no support that sick unemployed persons should receive their benefits unconditionally.

That unemployment is medicalized has also been acknowledged in the policy community (OECD, 2010). In response, activating sick unemployed persons has been high on the policy agenda. Numerous additional measures have been proposed and several countries have introduced reforms that specifically target this group (OECD, 2010; European Network of Public Employment Services, 2020). Considering that the group of sick unemployed persons is large and has proven difficult to integrate with existing activation measures, many countries have tried different measures to address this group. In Germany strengthening health promotion and rehabilitation for the unemployed has been a key goal of the Ministry of Labor and Social Affairs. In our article, we explored how citizens would support the inclusion of health-related measures in the mutual-obligation framework. We find that support for such measures is high even if they would be implemented as conditions for benefit receipt.

This limited opposition from the public to tightening obligations for the sick unemployed can be considered worrisome, since existing evidence on health-related interventions among unemployed persons is rather skeptical if and to what degree such measures lead to employment or improved health among this group (Hollederer, 2019; Hult et al., 2020). In any case, health-related obligations for unemployed persons would mean increasing pressure on a group that is already vulnerable in two respects.

### Limitations

The results of this study are subject to limitations. First, the experimentally varied vignette levels in our study measure the deservingness and social control perceptions emerging from chronic back pain and depression. Other operationalizations of control or illness, such as cancer or COVID-19, may lead to different assessments of deservingness. Nevertheless, our operationalization are plausible and common reasons for individuals' unemployment (Paul and Moser, 2009). Second, our vignettes do not consider all factors that could be relevant in real-life situations. For instance, gender, education, the length of the payment and withdrawal periods of unemployment benefit were not varied across the vignettes. While varying these factors could provide further insights, as long as they do not interact with the factors under study, our results remain valid (Auspurg and Gundert, 2015). Third, our study is not based on a random sample. Therefore, estimates and confidence intervals could be incorrect. However, the sampling frame of the YouGov panel is quite large and the quota and weighting procedures ensure that the demographic characteristics are close to population values. Moreover, existing studies evaluating online access panels also have shown that their results are in many cases similar to probability samples (Vehovar et al., 2016). Finally, our study is limited to one country. Since contextual factors are known to be relevant for welfare attitudes (Jeene et al., 2014; Buß et al., 2017), future research could investigate the extent to which our results can be generalized to other countries.

## Conclusions

Despite these limitations, our goal with this study was to contribute to a growing body of research demonstrating that medicalization theory can be fruitfully used in a quantitative research designs (Pattyn et al., 2013; Christiaens and Bracke, 2014; Buffel et al., 2015, 2017; Wong, 2016). Due to increasing conditionality of benefits (Dwyer, 2008) and institutionally forced disengagement from a broader social context toward more individual responsibility in dealing with reduced employability (Holmqvist, 2010), in many countries, sickness and disability now represent the only path to escape the activation logic (Holmqvist, 2009) or even to receive benefits (Wong, 2016). Particularly in the German system, where the barriers to accessing benefits are relatively high and a very strong expectation is attached to receiving benefits (Venn, 2012), we show that an sickness significantly lowers the acceptance of sanctions. This demonstrates how a sociological analysis based on medicalization theory provides an important, innovative perspective on research questions within the welfare state more broadly (Olafsdottir and Beckfield, 2011).

## Data Availability Statement

The raw data supporting the conclusions of this article will be made available by the authors, without undue reservation.

## Ethics Statement

The study involving human participants were reviewed and approved by the Council of Research Ethics (Ethics Council) of the University of Siegen and the Ombudsman System for the Safeguarding of Good Scientific Practice. The participants of the YouGov panel Germany provided their written informed consent to participate in this study.

## Author Contributions

All authors listed have made a substantial, direct, and intellectual contribution to the work and approved it for publication.

## Funding

This work was supported by the Support Network for Interdisciplinary Social Policy Research (FIS) of the German Federal Ministry of Labor and Social Affairs (Grant Number: Ia4-12141-1115).

## Conflict of Interest

The authors declare that the research was conducted in the absence of any commercial or financial relationships that could be construed as a potential conflict of interest.

## Publisher's Note

All claims expressed in this article are solely those of the authors and do not necessarily represent those of their affiliated organizations, or those of the publisher, the editors and the reviewers. Any product that may be evaluated in this article, or claim that may be made by its manufacturer, is not guaranteed or endorsed by the publisher.

## Footnotes

^1^Pursuant to Article 1 (2) of the German Dismissal Protection Act (Kündigungsschutzgesetz - KSchG), a dismissal may be socially justified if it is based on reasons relating to the person of the employee. Such a termination for personal reasons requires that the employee is (permanently) unable to perform the work owed by him/her under the employment contract due to his/her personal abilities and characteristics. In addition to other reasons, sickness is also to be understood under this, even if the employer must justify the termination beyond doubt.

^2^Even though a reduction of up to 100% goes beyond the current legal framework, the possibility of higher sanctions was not limited to a more severe breach of duty to obtain the most comparable results between the factor of missed appointments.

^3^We excluded participants who completed the survey in under 9.31 min (below the 33rd percentile).

^4^We highlighted that those descriptions were hypothetical, and respondents' decisions had no influence on real persons. We gave information about underlying theoretical implications and provided contact information of the research team for further inquiries.
